# Years-long stability of X10 expansion microscopy hydrogels

**DOI:** 10.1186/s13036-026-00781-3

**Published:** 2026-10-14

**Authors:** Eman Abbas, Ali H. Shaib, Mohamed A. Hassan, Silvio O. Rizzoli

**Affiliations:** 1 Department of Neuro- and Sensory Physiology, University Medical Center Göttingen, Göttingen, Germany; 2 Center for Biostructural Imaging of Neurodegeneration, University Medical Center Göttingen, Göttingen, Germany; 3 Cluster of Excellence “Multiscale Bioimaging: from Molecular Machines to Networks of Excitable Cells” (MBExC), University of Göttingen, Göttingen, Germany

**Keywords:** Expansion microscopy, Post-labeling, Post-fixation, X10 hydrogel

## Abstract

Expansion microscopy (ExM) is developing rapidly to explore biological structures, both towards nanoscale imaging and the wider-scale analysis of cells and tissues. ExM is based on embedding samples in swellable hydrogels, which then expand to enable superior microscopy performance with conventional microscopes. However, imaging precision is limited by the stability of the hydrogels, which are typically regarded as less robust than conventional samples. Here, we show that hydrogels based on *N*,* N*-dimethylacrylamide (DMAA) remain stable for several years, without a perceivable loss of structure. No special measures of stabilization are necessary for the gel matrix, but post-fixation is required to maintain the integrity of antibody labeling, when post-expansion labeling procedures are employed. Otherwise, antibodies slowly lose epitope binding over years, corrupting both signal intensity and resolution. We conclude that ExM hydrogels are substantially more robust than previously envisioned, enhancing signal intensity and enabling years-long performance, with minimal care. Moreover, this paves the avenue to re-imaging, inter-lab sample exchange, and retrospective analyses, which could be employed in medical applications ranging from diagnostic validation and biomarker discovery to comparative studies across time points or cohorts. This approach extends ExM from a single-use technique to a platform compatible with sustained experimental workflows.

## Graphical Abstract

**Figure:**
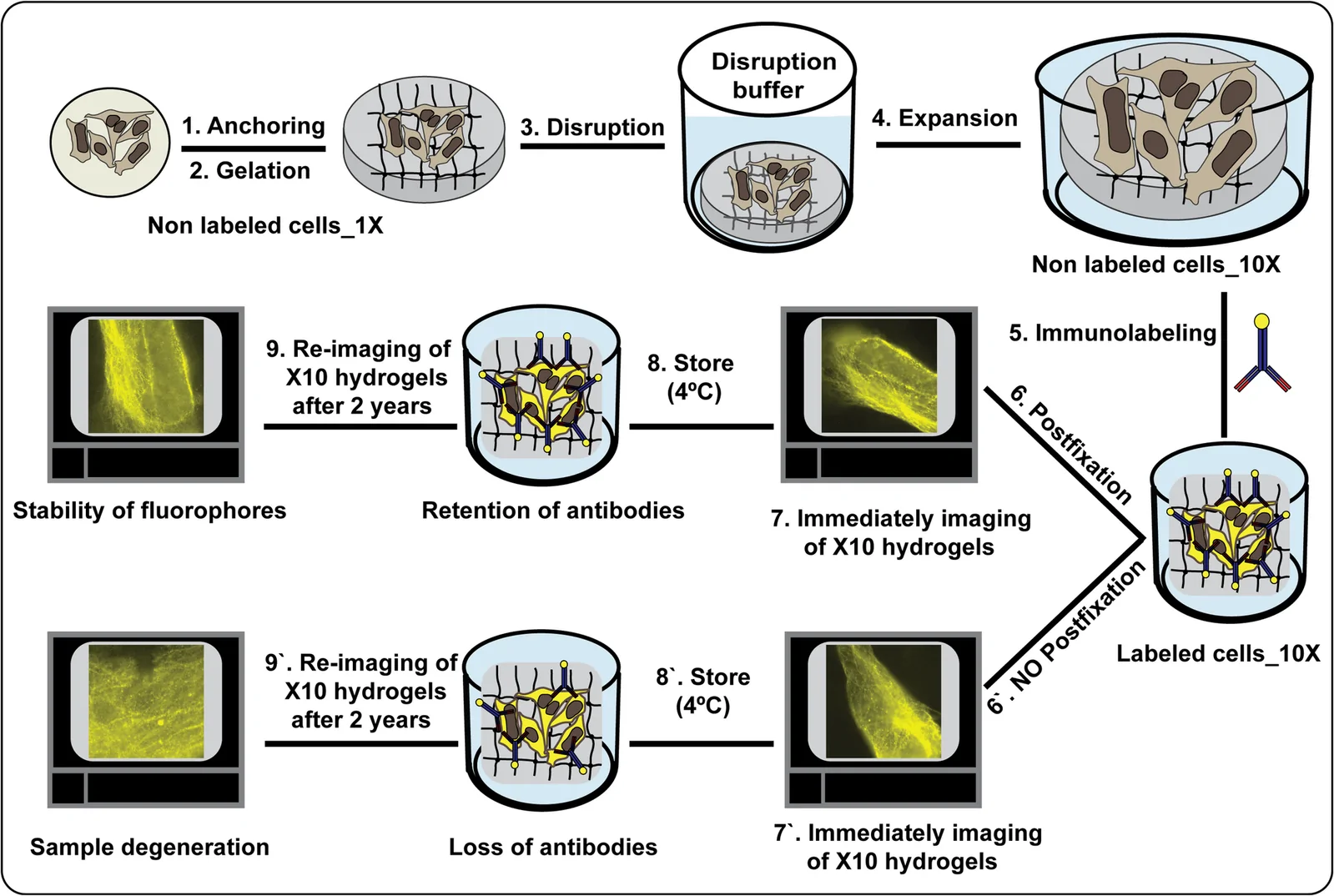

## Introduction

Expansion microscopy (ExM) is a pioneering imaging approach that enables high resolution imaging of biological samples, while employing conventional microscopes [1, 2]. It considers a stepwise protocol that entails the fixation and labeling of the desired target, which should be anchored to ensure the maintenance of spatial epitope retention [3, 4]. The samples are then embedded into a highly absorbent hydrogel, which greatly expands upon dialysis in water [4–6]. The application of advanced imaging technologies in ExM has recently garnered substantial attention in biology and medicine studies. For instance, the association of the ExM with super-resolution radial fluctuations (SRRF) imaging technique was applied to investigate the morphology of single proteins and analyze clinical samples of cerebrospinal fluid from Parkinson patients [5]. Another application, light microscope and connectomics (LICONN), was recently described by incorporating deep learning segmentation and connectivity analysis with ExM approach, enabling the analysis of individual neurons and their interactions in the brain [7].

Conventional biological specimens are commonly preserved by chemical fixatives, which stabilize tissue architecture through irreversible protein crosslinking, to conserve the structural integrity of the samples in its natural state [8]. Nevertheless, this type of preservation is not feasible in the expansion hydrogels. Expansion hydrogels are commonly composed of (AA), sodium acrylate (SA), and *N*,* N*'-methylenebisacrylamide (MBA), which are covalently linked through free-radical polymerization of vinyl groups. Acrylamide acts as core of the polymer matrix conserving the structural integrity, whereas MBA links the polymer chains, leading to the stabilization of the gel network. The addition of sodium acrylate to the medium could expand the gels to obtain high expansion factors [9–11].

A related formulation of the hydrogel has been designed by combining sodium acrylate with *N*,* N*-dimethylacrylamide (DMAA), instead of AA, to achieve expansion factors of around 10× [9, 10, 12]. Significantly, DMAA performs dual functions as a backbone of the gel and a self-crosslinker, when catalyzed with potassium persulfate [9]. Consequently, the generated DMAA-based hydrogel has more robust matrix resulting from uniform distribution of the crosslinking points, reducing the potential gel distortions triggered by crosslinking artifacts compared to bis-acrylamide-crosslinked hydrogels [10, 13]. Importantly, it is well known that homogenization is a crucial step in the expansion protocols, to achieve isotropic hydro-expansion, which is accomplished either via protease digestion or protein denaturation following the polymerization of hydrogel [14–16]. However, this step triggers label-loss issue, which is one of the predominant concerns of ExM [15]. Moreover, it was reported that certain dyes may be irreversibly destroyed due to highly radical-mediated polymerization process and inadequate covalent grafting into gels [9, 14, 15, 17].

Even though several studies have demonstrated the utilization of hydrogels in expansion microscopy to reveal the nanoscale structure and patterns of proteins [5, 6, 12], we now show the first report on sample stability and antibody retention over long time periods using DMAA-based and ultrastructure expansion microscopy (U-ExM) hydrogels, along with signal enhancement achieved through a post-labeling approach integrated with a post-fixation strategy.

We therefore hypothesized that applying chemical fixatives, such as paraformaldehyde (PFA) and glutaraldehyde (GA), to polymerized hydrogels should enhance signal intensity and preserve immunolabeled biological samples long-term, thereby optimizing the expanded sample preservation. To verify our hypothesis, we applied a post-expansion labeling approach to HEK293 cells, achieving an approximately 10× expansion factor and prolonged antibody retention over years. Moreover, we applied the same approach to neuronal cultures, which possess greater structural complexity and heterogeneity than HEK293 cells, and observed that the cellular structures embedded in the hydrogel remained highly stable over weeks. The resulting structures can be easily imaged by means of conventional fluorescence and advanced high-resolution microscopy.

## Methods

### HEK cells 293 preparation

Cultures of HEK 293 cells were grown on Poly-D-lysine-coated coverslips for 24 h at 37 °C in a humidified CO_2_ incubator. The attached cells were then fixed with 4% PFA prepared in PBS for 30 min at room temperature, followed by washing in PBS.

### Primary hippocampal culture preparation

Hippocampal cultures were prepared from postnatal day P0 Wistar rat pups as previously described [18]. Cells were plated onto poly D-lysine coated 12-well plates and incubated at 37 °C in a 5% CO_2_ humidified incubator. The cells were used for expansion between 14 and 18 DIV.

### X10 hydrogel preparation

Coverslips were processed for ExM, starting by the anchoring step to form covalent bonds between the proteins and the presumptive expanded gel. Acryloyl-X (cat. no. A-20770, Thermo Fisher Scientific), which serves as chemical anchoring agent, was diluted as 0.1 mg/ml in the buffer (pH 6) composed of 100 mM MES and 150 mM NaCl and then applied to the cells overnight at 4 °C. To obtain expansion factor up to 10×, the gel samples were developed as described by Truckenbrodt et al. [12]. Therefore, the monomers were prepared by mixing the *N*,* N*-dimethylacrylamide (DMAA, cat. no. 274135, Sigma-Aldrich) and sodium acrylate (SA, cat. no. 408220, Sigma-Aldrich) before being purged with nitrogen gas for 40 min at room temperature. The polymerization was initiated by potassium persulfate diluted by 0.4 mol% relative to the monomer concentration. Subsequently, the solution was purged with N_2_ gas for 15 min longer.

To accelerate the polymerization process, 40 µl of *N*,* N*,*N´N´*-tetramethyl-ethane-1,2-diamine (TEMED, cat. no. T7024, Sigma-Aldrich) were mixed into 10 ml solution, the mixture was placed on a parafilm and the samples were then placed on the gel solution in a humidified chamber at 37 °C for 1 h to complete the polymerization.

### Disruption and X10 expansion procedures

Polymerized hydrogels containing HEK Cells were incubated in denaturation buffer (pH 9.0) composed of 1.8% SDS in 100 mM Tris for 30 min. Afterward, the samples were incubated in the disruption buffer for 1.5 h, 2 h, 2.5 h and 3 h at 95 °C, and then washed twice with PBS. The gels were directly subjected to the expansion step using double deionized water until obtaining the maximum size of the hydrogels.

For neuronal cultures, the hydrogels harboring neuronal cultures were incubated in a denaturation buffer (pH 8.0) consisting of 5% Triton X-100 and 1% SDS in 100 mM Tris buffer (pH 8.0) for 30 min. The hydrogels were then incubated in the denaturation buffer for 1 h at 95 °C, and subsequently washed twice with PBS. Thereafter, the gels underwent the expansion step using double deionized water, until obtaining the ultimate hydrogel size.

### Post-expansion staining

Small pieces were incised from the gels and then incubated in the blocking solution (2% BSA in PBS) for 1 h at room temperature.

#### Indirect post-expansion labeling of expanded HEK 293 cells in gels with alpha tubulin

The gels were incubated with alpha tubulin (cat. no. 302211, SYSY) as a primary antibody at a dilution of 1:350 in 2% BSA in PBS overnight at 37 °C in a humidified chamber, the gels were washed 3 times with PBS for 5 min each. Later, secondary antibody Alexa Fluor 546 (cat. no. A-11030, Invitrogen) was diluted as 1:250 in 2% BSA in PBS and then applied to the hydrogels before being incubated at 37 °C for 3 h in a humidified chamber.

#### NHS-ester staining

To investigate the expanded HEK293 in DMAA-based hydrogels in the absence of antibody labeling, we post-labeled the cells with 20 mg/ml of NHS-ester fluorescein (cat. no. 46409, Thermo Fisher Scientific), in PBS, at pH 7.4 overnight at 4 °C. Subsequently, the hydrogels were rinsed three times in PBS and expanded in double deionized distilled water.

#### Direct post-expansion labeling of expanded astrocytes in gels with GFAP nanobodies

For this staining procedure, we used two types of GFAP nanobodies conjugated with distinct fluorophores. Accordingly, the gels were incubated with either GFAP nanobodies conjugated with the Atto 542 fluorophore (cat. no. N3805-GFAP, custom conjugation to Atto542, NanoTag Co.) or conjugated with the Alexa Fluor 633 fluorophore (cat. no. N3805-GFAP, custom conjugation to Alexa Fluor 633, NanoTag Co.), as a primary labeled nanobodies. Both nanobodies were applied at a dilution of 1:350 in 2% BSA in PBS, for 2 h at 37 °C in a humidified chamber. Subsequently, the gels were washed 3 times with PBS for 5 min each.

#### Preparation and post-labeling using acrylamide/bis-acrylamide-based hydrogels

We prepared ultrastructure expansion microscopy (U-ExM) gels, an acrylamide/bis-acrylamide-based formulation, as previously described, with slight modifications [19–21], for post-labeling of cells, followed by the post-fixation approach with PFA/GA.

Towards this end, fixed neurons were incubated in the anchoring solution (2% acrylamide, 1.4% formaldehyde) overnight at RT. Afterward, neuronal cells were rinsed 3 times with PBS, and then subjected to the gelation step by the addition of the monomer solution (MS) [19% (wt/wt) sodium acrylate, 10% (wt/wt) acrylamide, and 0.1% (wt/wt) N, N’-methylenbisacrylamide in PBS], with 0.5% (wt/v) TEMED and 0.5% (wt/v) APS added directly before use. Following polymerization, gels were incubated in a denaturation buffer (pH 9.0) containing 50 mM Tris, 200 mM NaCl, and 200 mM SDS for 30 min. The hydrogels were then incubated in the denaturation buffer at 95 °C for 1 h and subsequently washed twice with PBS. Following this, gels were subjected to the expansion step using double deionized water, followed by incubation with the blocking solution (2% BSA in PBS) for 1 h at RT. Afterward, hydrogels were stained employing indirect labeling using TOM20 (cat. no. 11802-1-AP, Proteintech), and GM130 (cat. no. 610822, BD Biosciences) at 37 °C overnight as primary antibodies (1:300 in 2% BSA) in a humidified chamber. The gels were then washed 3 times with PBS-T [0.2% (v/v) Tween in PBS] for 5 min each and then incubated with AF546 (cat. no. A-11030, Invitrogen) and Abberior STAR 635P (cat. no. ST635P-1002-500UG, Abberior) as secondary antibodies (1:350 in 2% BSA for 2 h at 37 °C).

Other gels were stained directly with nanobodies for PSD95 (conjugated to Cy3, cat. no. N3805-GFAP, custom conjugation to Alexa Fluor 633, NanoTag Co.) and synaptotagmin 1 (cojungated to STAR 635P, cat. no. N3805-GFAP, custom conjugation to Alexa Fluor 633, NanoTag Co.) nanobodies. Both nanobodies were applied at a dilution of 1:350 in 2% BSA in PBS for 2 h at 37 °C in a humidified chamber. Following that, gels were washed 3 times with PBS-T for 5 min each.

#### Division of the post-staining gel into groups and expansion of the gels

The hydrogels (DMAA-based and U-ExM hydrogels) were rinsed twice in PBS and then divided into two groups. The first group was subjected directly to the expansion process using double deionized water, while the second group of hydrogels was exposed to post-fixation step using a mixture of 1% glutaraldehyde (GA) in 4% paraformaldehyde (PFA) for 20 min at room temperature under hood. Following this, the post-fixed hydrogels were subjected to extensive washing with PBS to ensure complete removal of free aldehydes, and then the hydrogels underwent the expansion process using double deionized distilled water. All gel samples were stored in double deionized distilled water at 4 °C, without changes over the preserved time, and were imaged at different time intervals. The samples were maintained in conventional, plastic cell culture dishes, without strict aseptic operation.

### Swelling profile of hydrogels

To estimate the swelling profiles of untreated and PFA/GA-treated DMAA-based hydrogels, we immersed the specimens in double-distilled water for predetermined time intervals ranging from 5 to 60 min. At each time point, gels were taken out, and all evaluations were conducted in triplicate. The swelling ratio was calculated with reference to the initial mass of the non-swollen hydrogel using the following equation:

$$\mathrm{Swelling}\:\mathrm{ratio}\:(\%) = [(\mathrm{St}-\mathrm{Si})/\mathrm{Si}]\times100$$

where St and Si indicate the mass of swollen hydrogel at a respective time and the initial mass of non-swollen hydrogel, respectively.

### Imaging and microscope systems

To image the gels, they were placed in the gel imaging chamber and visualized employing a conventional fluorescence microscope and a confocal laser scanning microscope. Single optical plane fluorescent overviews were acquired in xy with an Olympus IX71 fluorescence microscope equipped with 20× objective lens (UPlanFL N 20x/0.50,∞/0.17/FN26.5) and a pixel size of 322.5 nm. All confocal and STED images were acquired on a Leica STELLARIS 8 microscope (Leica Microsystems, Wetzlar, Germany) equipped with a HC PL APO CS2 100×/1.40 NA oil-immersion objective lens. An immersion oil objective with a refractive index of 1.518 was used. The White Light Laser (WLL) was used as the main excitation source.

3D overview images of α-tubulin labeled with Alexa Fluor 546 in expanded HEK293 cells were acquired in xyz mode using bidirectional scanning. Images were acquired as 3D z-stacks with 512 × 512 pixels per optical section and up to 28 optical sections. The physical image size was 11.62 × 11.62 × 4.93 μm, corresponding to a voxel size of 20 × 20 × 180 nm. The pixel dwell time ranged between 1.025 and 2.075 µs. The pinhole was set to 1 Airy unit, corresponding to 151.6 μm. Frame averaging, line averaging, and frame accumulation were set to 1, and line accumulation was set to 2. Excitation was performed with the 557 nm WLL laser line at 30% intensity, with the WLL operated at 85% maximum power. Detection was performed using the HyD S3 detector in photon-counting mode. The detector gain was not applicable in photon-counting mode, and the offset was set to 0.

For 3D-STED imaging of Alexa Fluor 546 α-tubulin samples, depletion was performed using the 660 nm pulsed STED laser. The STED 660 laser was active at 85% output power in the system settings, and the 660 nm laser line was set to 20% intensity in the acquisition settings. The 557 nm WLL excitation line was set to 30% intensity. TauSTED detection was enabled using the HyD S3 detector in photon-counting mode, with TauStrength set to 100. The theoretical pixel size was 20 nm in x and y. The STED beam path included STED Beam 1, CleanUp 660, SD 660, and Vortex 650. The STED Phase Filter Beam 2 was set to blocking. The notch filter wheel contained NF 660, and the polarization filter wheel contained NF 448/514/561. The X2 lens changer was set to CS2 UV Optics 2.

Acquisition parameters were standardized across samples. 2D or 3D overview confocal images of expanded cells were acquired in xyz mode using unidirectional scanning. Images were acquired as 3D z-stacks or single planes with 512 × 512 pixels per optical section. The pixel dwell time was 7.6125 µs. The pinhole was set to 1 Airy unit, corresponding to 151.6 μm. Frame averaging, line averaging, and frame accumulation were set to 1, and line accumulation was adjusted to 3. WLL operated at 85% of maximum power with detection via HyD detectors (S1, S3, or X4) in photon-counting mode. The detector gain was not applicable in photon-counting mode, and the offset was set to 0.

Expanded HEK cells labeled with NHS-ester fluorescein were imaged as 3D z-stacks using 491 nm laser lines at 18% intensity. Expanded astrocytes (GFAP labeled with Atto 542 or Alexa Fluor 633) were imaged as 3D z-stacks using 542–633 nm laser lines at 22% intensity. Expanded neurons were imaged using 633 nm laser line at 20% intensity for TOM20 and synaptotagmin 1 labeled with STAR 635P, and 557 nm laser line at 15% intensity for GM130 labeled with Alexa Fluor 546 and PSD95 labeled with Cy3.

ONE microscopy images were acquired in xyt mode using the Tandem Scanner at 12,000 Hz with unidirectional scanning. Each acquisition consisted of 1500 frames of 128 × 128 pixels, acquired over 59.31 s with a frame interval of 40 ms. The physical field of view was 11.62 × 11.62 μm, corresponding to a pixel size of 90 × 90 nm in the expanded sample. The microscope-reported pixel dwell time was 0.2065 µs. The zoom factor was 10. The pinhole was set to 1 Airy unit, corresponding to 151.6 μm. Frame averaging, line averaging, and frame accumulation were set to 1, and line accumulation was set to 3. The WLL was operated at 85% maximum power. The notch filter wheel was set to Empty, and the polarization filter wheel contained NF 448/514/561. Detector gain, offset, laser intensity, and detection settings were kept identical between the compared samples.

For each direct comparison, including newly prepared versus stored expanded HEK293 cells, neurons, and astrocytes, the same optical acquisition settings were applied to the respective samples. This included the same microscope, objective, scan mode, scan speed, pixel dwell time, voxel size, laser line and laser intensity, detector, detection window, detector gain/offset settings, averaging and accumulation settings, and STED depletion settings where applicable.

## Results

**Fig. 1:**
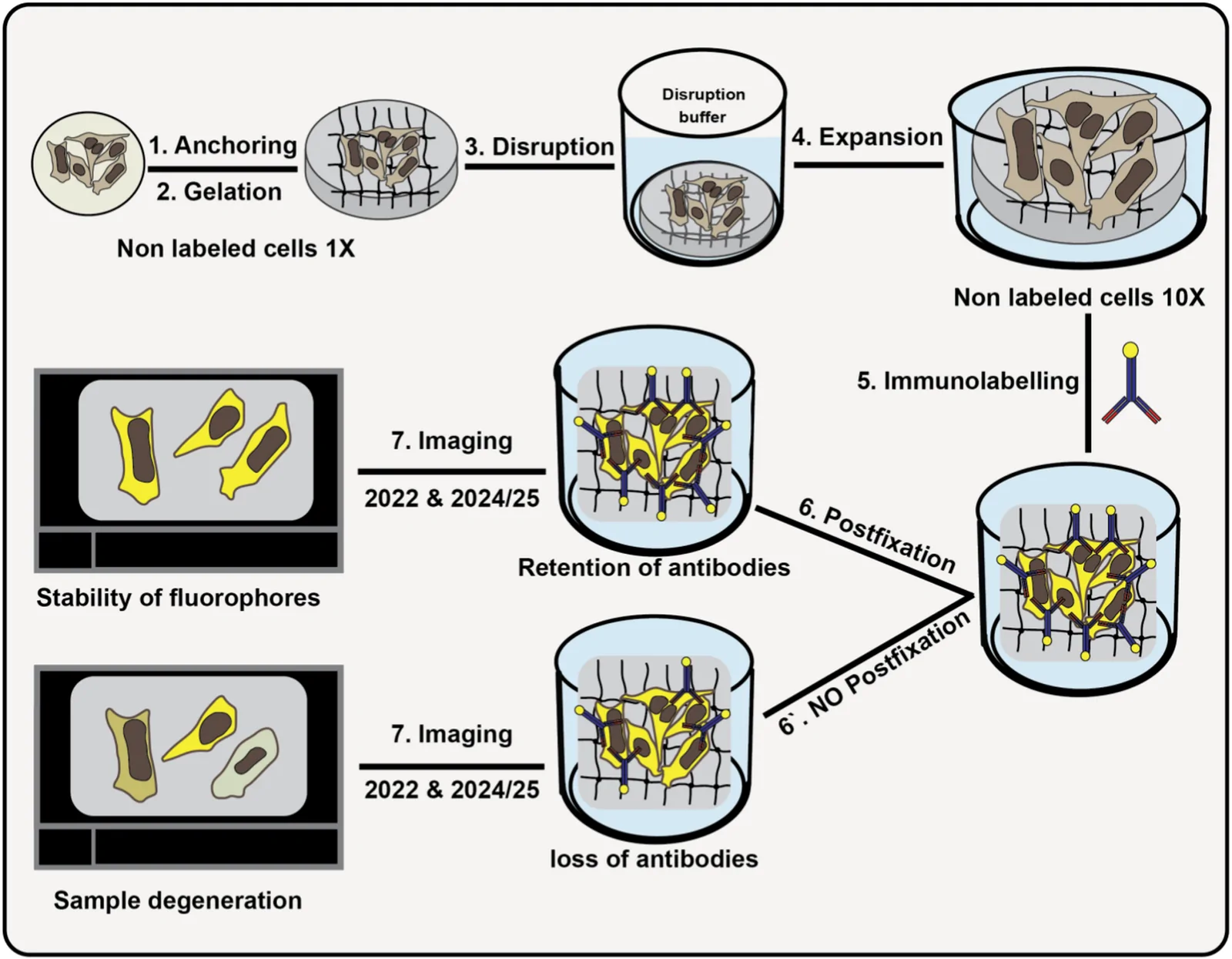
Workflow of X10 expansion microscopy, illustrating post-labeling and post-fixation approaches performed in this study

To explore the long-term stability of the gels, we relied on microtubules, a commonly imaged protein structure in fluorescence microscopy. We immunostained the expanded HEK293 cells with alpha tubulin as a primary antibody and secondary antibody conjugated to Alexa Fluor 546, followed by post-fixation of hydrogels in a mixture of 4% PFA and 1% GA, while other hydrogels were not subjected to post-fixation procedures. Figure 1 delineates the workflow of X10 expansion procedures conducted in this study.

**Fig. 2:**
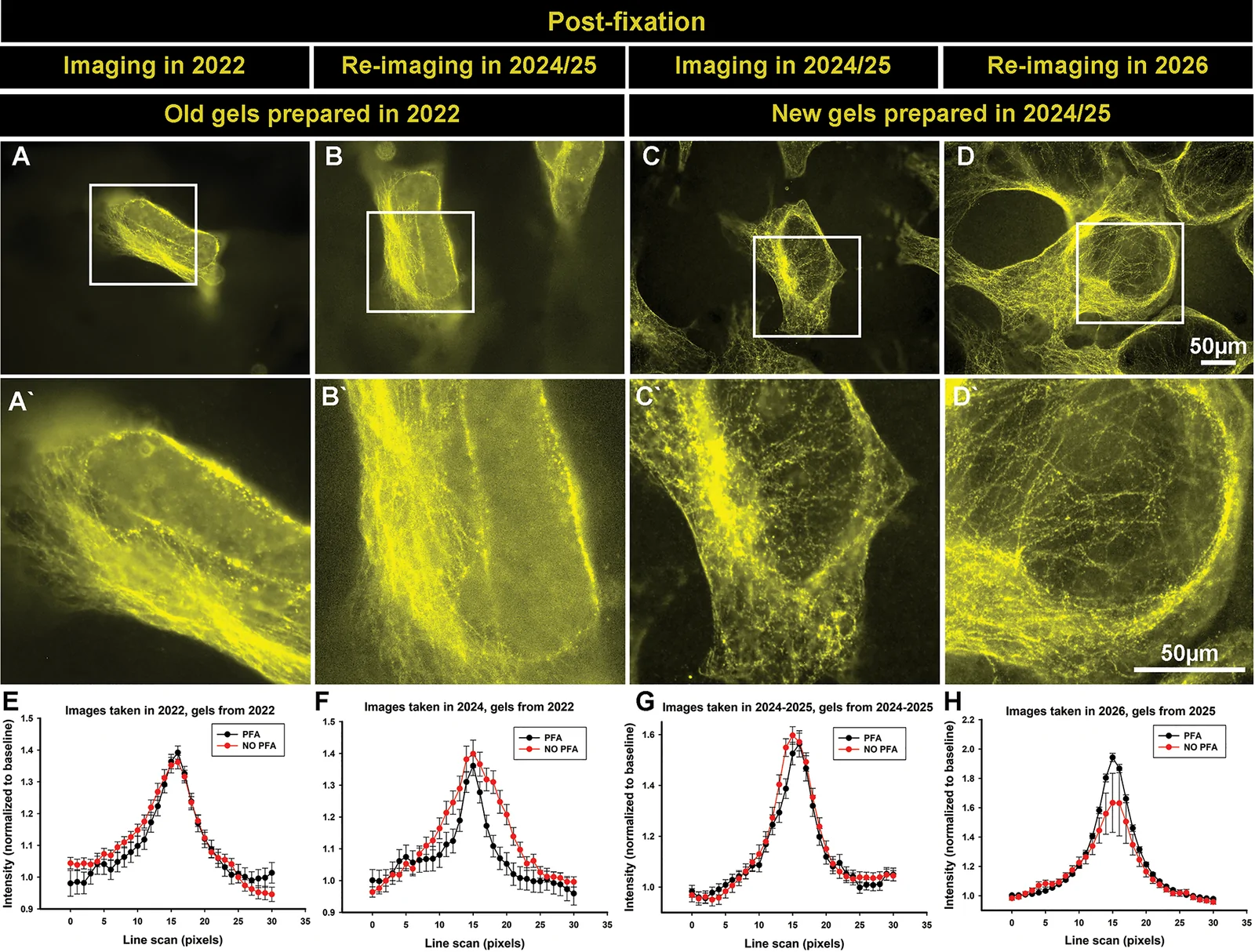
Post-fixation preserves antibody labeling and structure over years. (**A**-**D**) 2D Epifluorescence overviews of post-labeled alpha tubulin microtubules in expanded HEK293 cells. (**A**) Post-fixed hydrogel prepared and imaged in 2022 and (**B**) the same hydrogel was re-imaged in 2024/25 after storage at 4 °C. (**C**) Newly prepared and imaged post-fixed hydrogel in 2024/25 and (**D**) the same hydrogel was re-imaged in 2026 after storage at 4 °C. (**A`**-**D`**) Insets show higher magnification views of representative regions. (**E**-**H**) Line scan intensity profiles across microtubules from the corresponding conditions, comparing post-fixed and non-post-fixed hydrogels. Post-fixation maintains fluorescence intensity and spatial definition over time, whereas untreated samples show reduced signal stability. *N* = 50 cells from five independent hydrogels were analyzed with a pixel size of 322.5 nm

We examined the staining quality of the cytoskeleton by imaging single plan overviews of expanded HEK293 cells employing a conventional fluorescence microscope. As shown in Fig. 2, the overview images and the enlarged inserts depict the specific post-expansion labeling patterns of microtubules in expanded HEK cells.

**Fig. 3:**
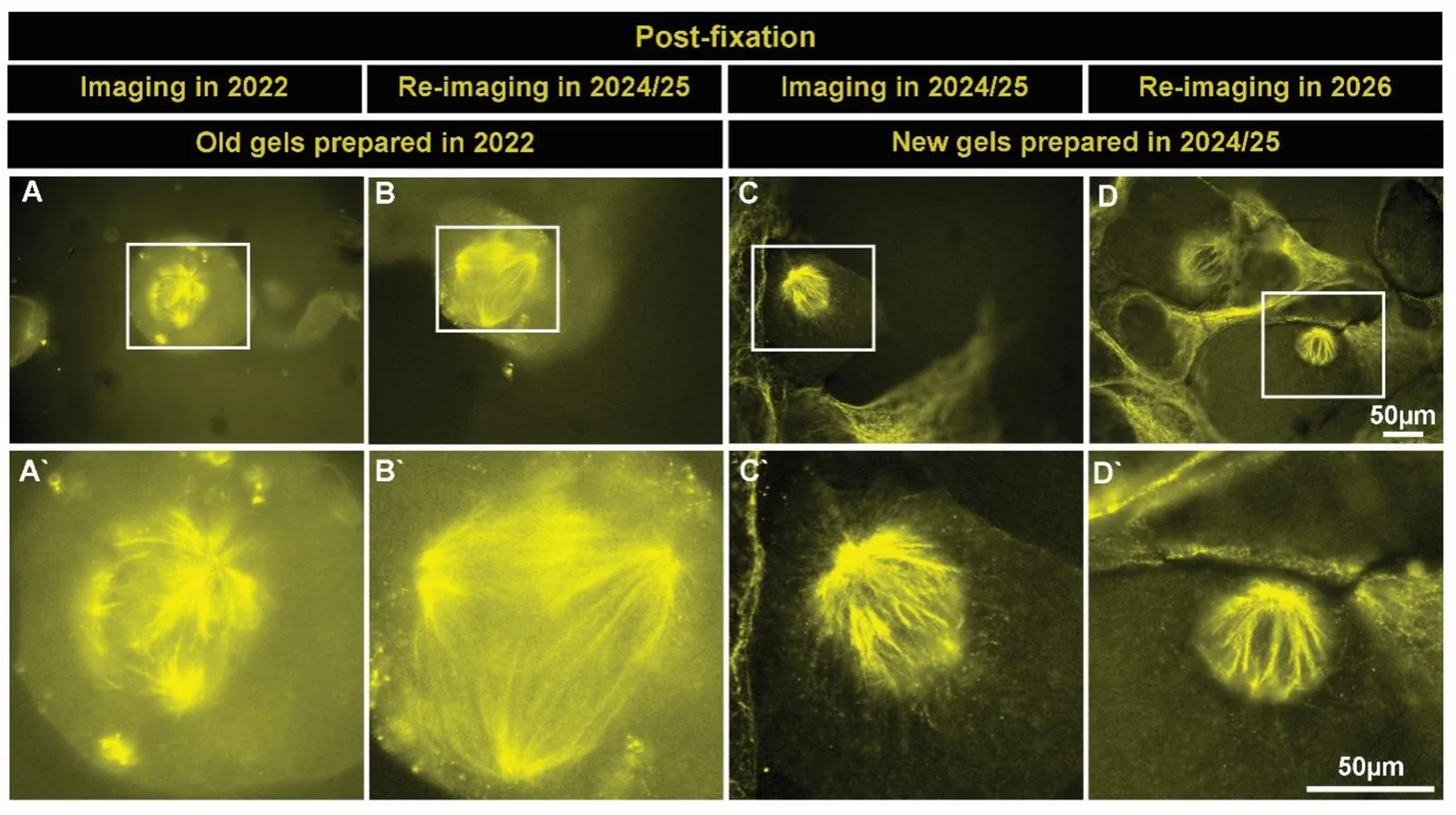
(**A**-**D**) 2D fluorescence micrographs of post-labeled alpha tubulin spindle microtubules in expanded HEK293 cells. (**A**) Post-fixed hydrogel prepared and imaged in 2022 and (**B**) the same hydrogel was re-imaged in 2024/25 after storage at 4 °C. (**C**) Newly prepared and imaged post-fixed hydrogel in 2024/25 and (**D**) the same hydrogel was re-imaged in 2026 after storage at 4 °C. (**A`**-**D`**) Insets show higher magnification views of representative regions. *N* = 12 cells from five independent hydrogels were analyzed

Furthermore, Fig. 3 illustrates the network of filaments that develop microtubule features, along with the distinct structure of spindle fibers in some dividing cells, indicating good-quality labeling.

To investigate the effect of post-fixation treatment on expanded post-labeled HEK cells, we prepared two groups of hydrogels. The first group was treated with PFA and GA, and the second group did not expose to post-fixation treatment. Our results demonstrated that post-fixation treatment could boost the consistency of signal intensity without impeding the expansion of the hydrogel.

As we started this project in 2022 and the gels were stored at 4 °C, we re-imaged the same gels in 2024/25 by means of the same conventional epifluorescence microscope (Fig. 2A-B). Interestingly, we found that the gels are still working, displaying a good-quality. Remarkably, the recent fluorescent images of the old gels subjected to post-fixation, were captured in 2024/25, exhibited reasonable staining and antibody retention capacity, compared to those imaged in 2022. Additionally, we detected spindle microtubule organizations in some mitotic cells, implying increased stability of the samples (Fig. 3A-B).

To compare the images of old gels with newly prepared gels, we conducted the same experiments in 2024/25, following the same parameters and imaged the new gels applying similar imaging parameters as presented in Figs. 2C and 3C.

To further ensure the reproducibility of our findings, we stored the newly prepared gels in 2024/25 in the fridge and re-imaged in 2026 employing the fluorescence microscope applying the same parameters. Expectedly, we could visualize the structure of the cytoskeleton and spindle fibers, highlighting the higher staining quality and antibody retention achieved through the post-fixation treatment compared, to the gels prepared and imaged in 2024/25 (Figs. 2C-D and 3C-D). Moreover, the quantitative analyses revealed that the fluorophore intensities in the gels re-imaged in 2024/25 and 2026 are comparable to those prepared and imaged in 2022 and 2024/25 (Fig. 2D-H).

**Fig. 4:**
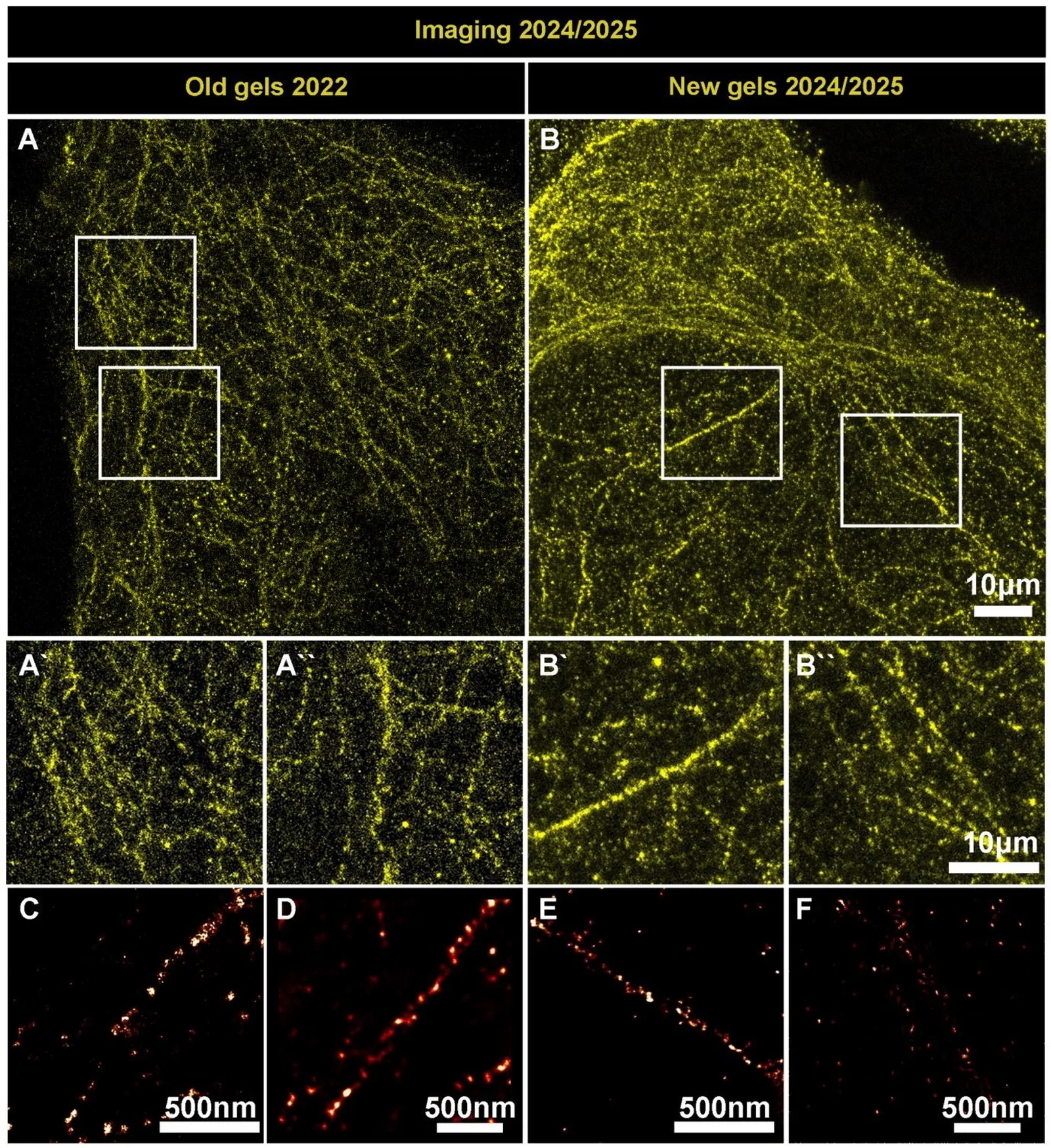
Microtubule architecture remains intact in post-fixed X10 hydrogels over multiple years across imaging modalities. (**A** and **B**) Confocal images of post-labeled alpha tubulin microtubules in expanded HEK293 cells from a hydrogel prepared in 2022 and re-imaged in 2024/25 (**A**), compared to a newly prepared hydrogel imaged in 2024/25 (**B**). (**A`**-**B``**) Higher magnification views of the indicated regions. (**C** and **E**) STED images and (**D** and **F**) ONE microscopy images of the same respective conditions demonstrate comparable microtubule organization and fine structural preservation. Confocal images were selected from more than 5 cells, while STED and ONE images were selected from more than 10 cells, taken from five independent hydrogels

To delve into the organization of alpha tubulin labeled microtubules, we scanned the old and newly developed hydrogels by means of confocal microscopy, thereby validating our procedures as delineated in Fig. 4. The confocal, STED, and ONE images evidently revealed the organization of the cytoskeletal molecules in the old gels prepared in 2022 that were re-imaged in 2024/25 compared to recently prepared gels in 2024/25. Similar results were obtained for both old and newly prepared gels (Figs. 4A-F).

**Fig. 5:**
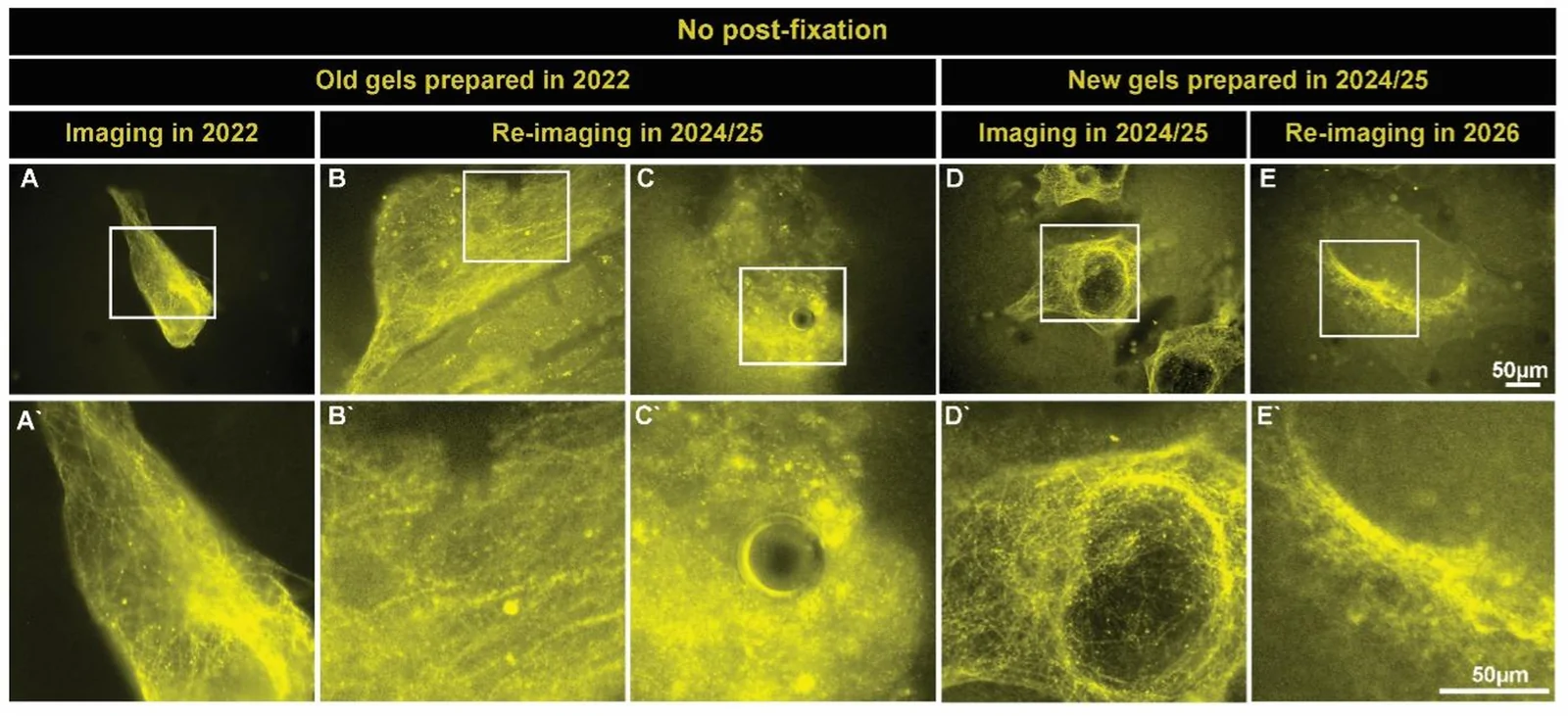
Absence of post-fixation resulted in progressive loss of labeling and structural degradation in X10 hydrogels. (**A** and **A´**) Alpha tubulin post-labeled microtubules in expanded HEK293 cells from a non-post-fixed hydrogel imaged in 2022. (**B** and **B`**) The same hydrogel re-imaged in 2024/25, showing substantial signal loss and reduced structural characterization. (**C** and **C`**) Severe degeneration was observed in some non-post-fixed samples after long-term storage. (**D** and **D`**) Newly prepared non-post-fixed hydrogel imaged in 2024/25, showing well-recognized microtubule structure, while (**E** and **E`**) the same hydrogel re-imaged in 2026 demonstrated progressive decrease of the signal and the structural details, thereby corroborating our findings. *N* = 30 cells from five independent hydrogels were analyzed

The hydrogels of the second group that were not treated with the mixture of PFA and GA displayed good resolution images with the typical structure of alpha tubulin microtubules when imaged in 2022 (Fig. 5A). However, the quality of the images dramatically diminished over the years, exhibiting poor alpha tubulin-labeled microtubules with noticeable disintegration of cytoskeletal conformation, as shown in the re-imaged gels in 2024/25 (Fig. 5B). As expected, we found complete degeneration in some hydrogel samples untreated with PFA and GA (group 2), which could be attributed to mold-induced degradation of the samples (Fig. 5C). Notably, the newly prepared gels, free of post-fixing agents, demonstrated comparable structures to the gels prepared and imaged in 2022 (Fig. 5D).

To validate our findings, we preserved the gels prepared in 2024/25 in the fridge and re-imaged them in 2026, using the same imaging parameters. As anticipated, it was significantly challenging to detect distinct alpha tubulin cytoskeletal structures, although no mold growth was observed on these gels. This indicates the significance of the post-fixation step in maintaining the structure of the proteins and reducing the loss of the antibodies over a long duration (Fig. 5D and E). These findings clearly indicate the positive effect of post-fixation on the long-term preservation of expanded HEK293 cells labeled with alpha tubulin. The experiments necessitate preservation of the gels for long-term up to years, before re-imaging using several replicates to achieve the goal of this study. The typical results were obtained, which are represented by the results shown and analyzed in this work.

**Fig. 6:**
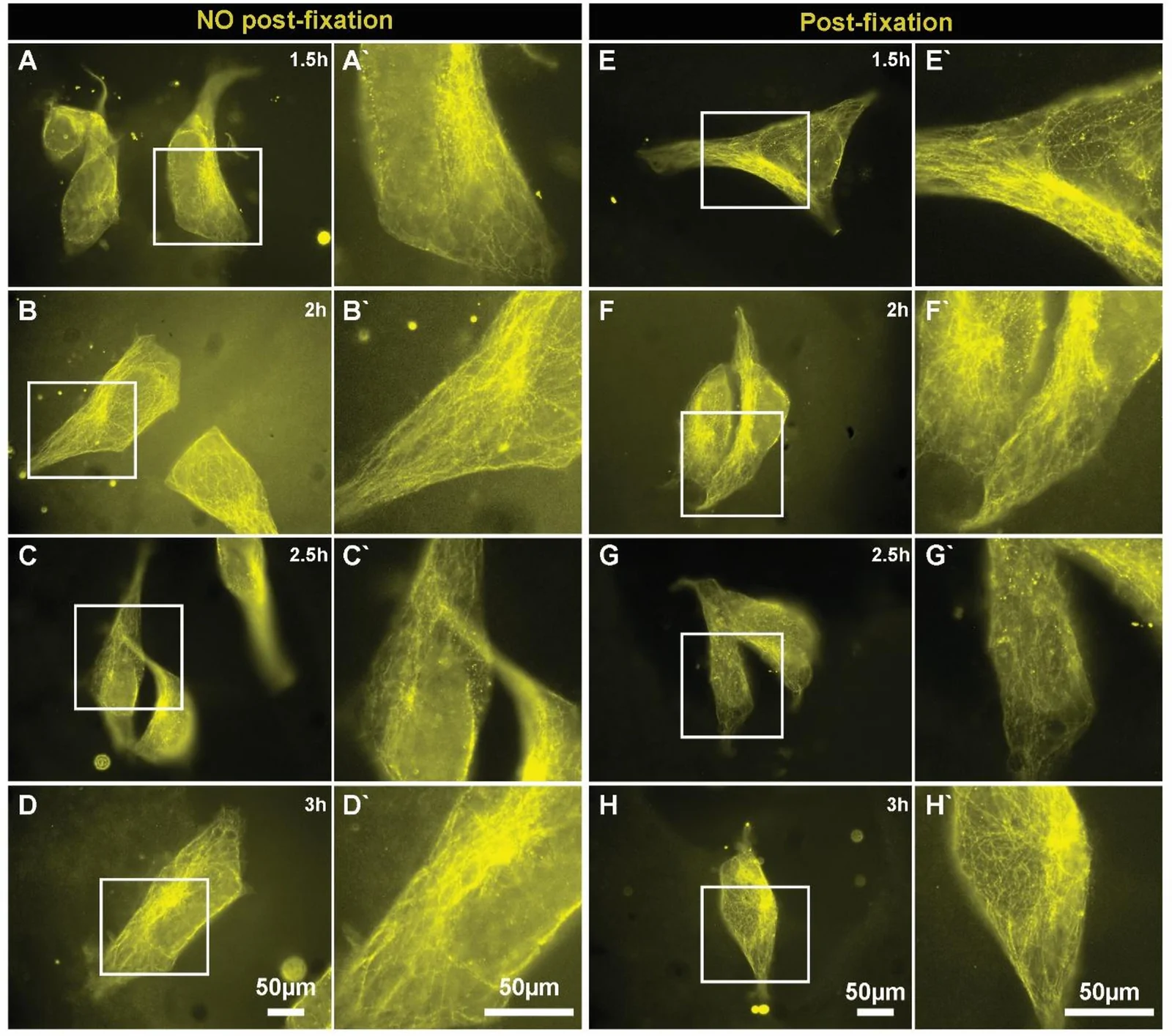
Disruption time and post-fixation do not significantly alter the size of expanded HEK293 cells. Expanded HEK293 cells labeled for alpha tubulin following disruption times of 1.5 h, 2 h, 2.5 h, and 3 h. (**A**-**D** and **A`**-**D`**) Post-fixed hydrogels. (**E**-**H** and **E`**-**H`**) Non-post-fixed hydrogels. Across all conditions, expanded cell dimensions remain comparable, indicating that post-fixation does not interfere with hydrogel expansion. The images shown were selected from 40 imaged cells taken from five independent hydrogels

We also determined the size of the cells in both groups, including no post-fixed and post-fixed gels, which underwent different softening times (1.5 h, 2 h, 2.5 h, and 3 h). The figures demonstrate that expanded HEK cells expressing alpha tubulin in both groups showed comparable size over various disruption times (Fig. 6). Our findings indicate that the post-fixation associated with different disruption times treatment shows no significant effect on the expansion of HEK cells.

**Fig. 7:**
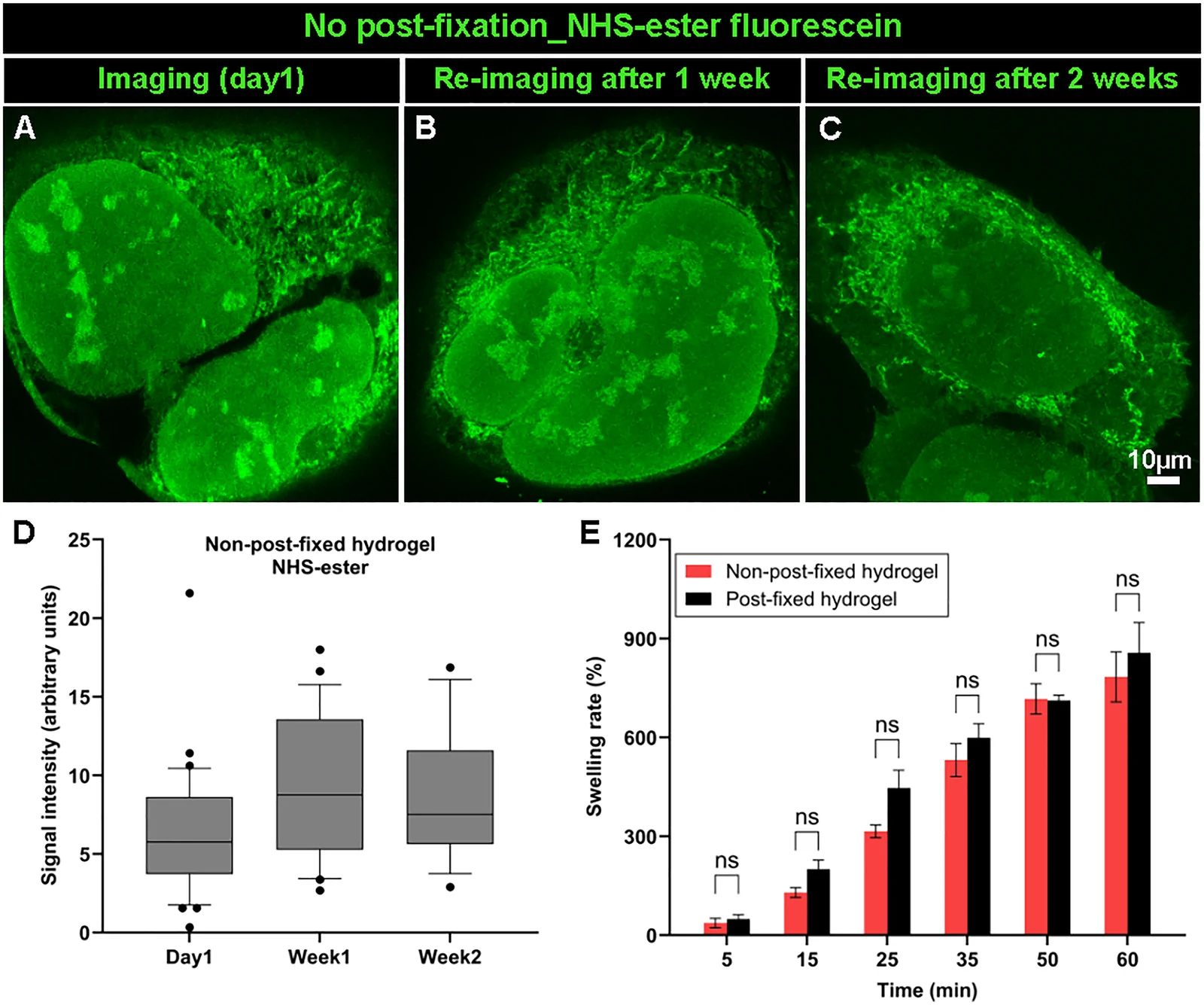
Stability of NHS-ester labeling and swelling profiles of DMAA-based hydrogels. (**A**-**C**) Confocal images of NHS-ester fluorescein-labeled and expanded HEK293 cells, in the absence of antibody labeling and post-fixation, acquired one day, one week, and two weeks after expansion. (**D**) Quantitative analyses reveal no diminution in signal intensity over time, signifying that the fluorescent dye remains stable, without fixation. *N* = 35 cell areas for day 1, 23 for week 1, and 15 for week 2. (**E**) Swelling rate profiles of non-post-fixed (No PFA/GA) and post-fixed (PFA/GA) DMAA-based hydrogels at predetermined time intervals. The results revealed no significant difference in swelling ratios between the two groups, suggesting that post-fixation does not compromise the hydrogels’ swelling properties. Data are shown as mean ± SEM (*N* = 3)

To assess the stability of the fluorescent dye, independent of antibody binding, we performed control experiments using NHS-ester fluorescein in the absence of antibody labeling and post-fixation. NHS-ester fluorescein labels the amine groups on the proteins and peptides in the gel. The NHS-ester reaction results in a covalent bond between the proteins and fluorescein, implying that the fluorescein signal should remain unchanged during long-term storage, since the covalently bound fluorescein molecules cannot diffuse away from the gel-bound proteins, irrespective of fixation. Figure 7A-C depicts confocal images obtained one day, one week, and two weeks after expansion of the same gels, respectively. The images and quantitative analysis revealed no reduction in signal intensity over the observation period (Fig. 7A-D).

Additionally, we investigated the swelling profiles of non-post-fixed and post-fixed DMAA-based hydrogels, to determine whether post-fixation modifies the gel, and thus alters the swelling ratio. As shown in Fig. 7E, the swelling profile data, across different time intervals, demonstrated no significant differences between non-post-fixed and post-fixed hydrogels, indicating that the gel matrix maintained its integrity after exposure to the PFA/GA fixative.

**Fig. 8:**
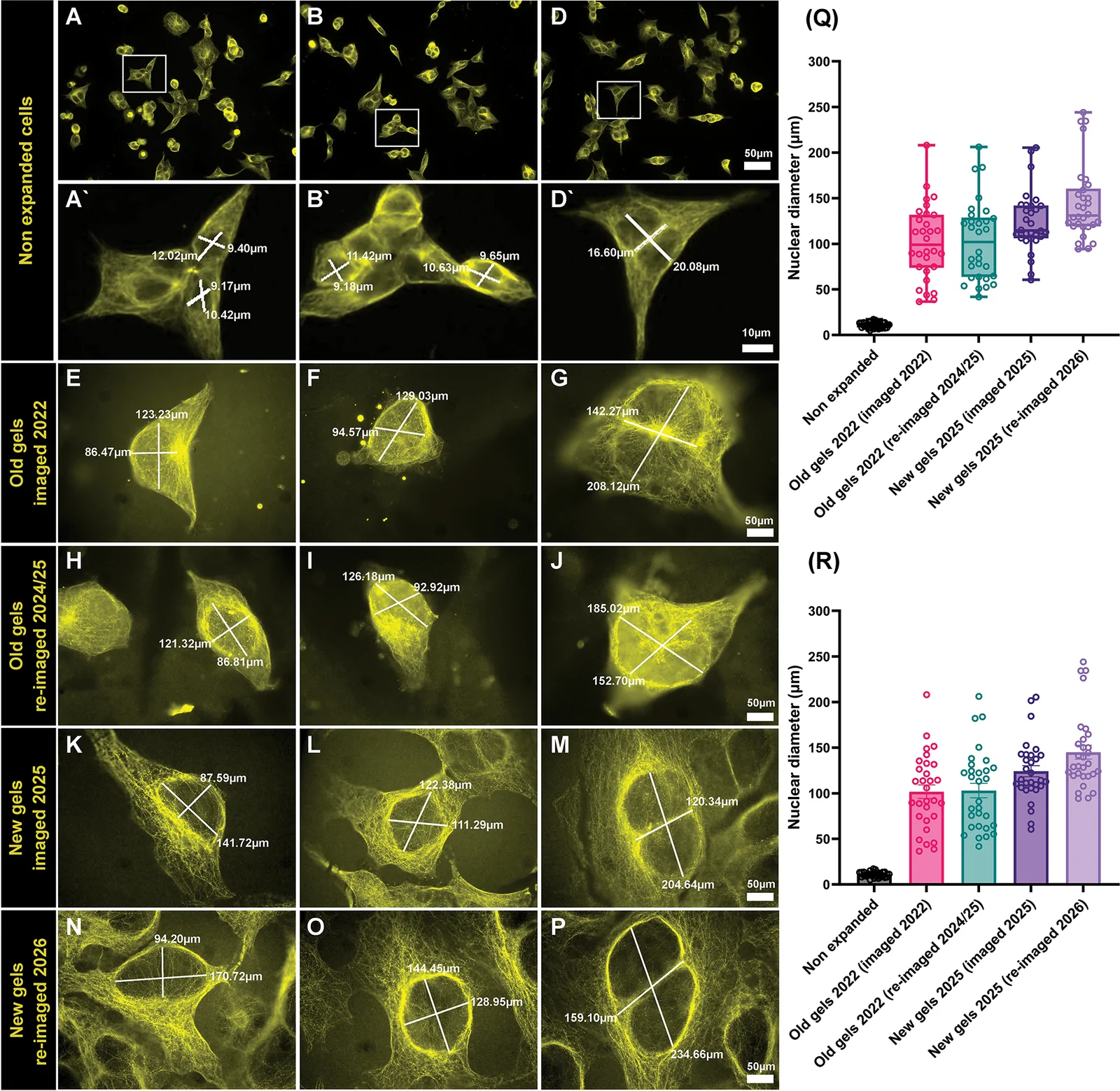
Quantitative assessment of X10 expansion factor in old and newly prepared hydrogels. (**A**-**D** and **A`**-**D`**) Non-expanded HEK293 cells labeled with alpha tubulin. (**E**-**G**) Expanded cells from hydrogels prepared and imaged in 2022 and (**H**-**J**) the same hydrogels re-imaged in 2024/25. (**K**-**M**) Newly prepared hydrogels imaged in 2024/25 and (**N**-**P**) the same hydrogels re-imaged in 2026. Nuclear diameters were measured to estimate expansion factors relative to non-expanded controls. All images were acquired with the same fluorescence microscope (20× objective lens). (**Q** and **R**) Quantification shows consistent approximately 10-fold expansion across all conditions, reported as mean ± SEM. *N* = 20 cells from five independent hydrogels were analyzed

Additionally, we measured the nuclear diameters of alpha tubulin-stained expanded HEK cells in the old and new gels treated with a post-fixation mixture of PFA and GA relative to measurements with non-expanded cells that served as controls as depicted in Fig. 8. Nuclear diameter measurements and quantitative analyses displayed expansion factors of ~ 10× in expanded cells compared to their respective non-expanded controls. Notably, the findings revealed no substantial differences between the old gels imaged in 2022 and 2025 and the newly prepared gels imaged in 2025.

**Fig. 9:**
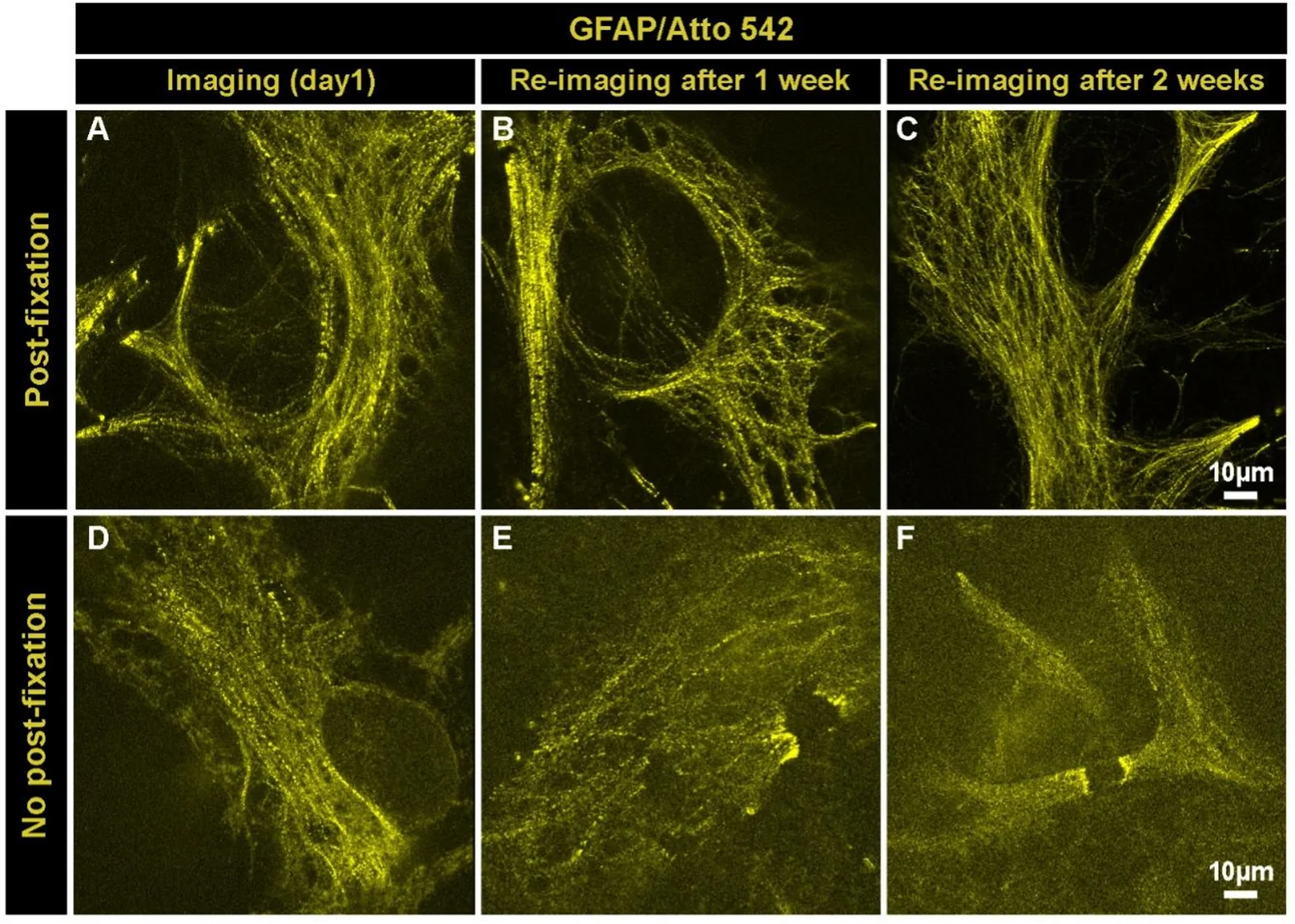
Overviews astrocytes labeled after expansion with GFAP nanobodies conjugated with Atto 542. (**A**) Post-fixed hydrogel imaged one day after post-expansion labeling, showing well-defined GFAP filaments, (**B**) the same post-fixed hydrogel after one week of storage at 4 °C, showing the distinct features of GFAP filaments, and (**C**) the same post-fixed hydrogel re-imaged after two weeks of storage at 4 °C, displaying the preservation of fluorescence intensity. (**D**) Non-post-fixed hydrogel imaged one day after post-expansion labeling, exhibiting the typical GFAP filament structure, (**E**) the same non-post-fixed hydrogel re-imaged after one week of storage at 4 °C, showing a noticeable reduction in fluorescence intensity, and (**F**) the same non-post-fixed hydrogel re-imaged after two weeks of storage at 4 °C, demonstrating substantial loss of signal and structural properties. *N* = 60 cells imaged from five independent post-fixed and non-post-fixed hydrogels

**Fig. 10:**
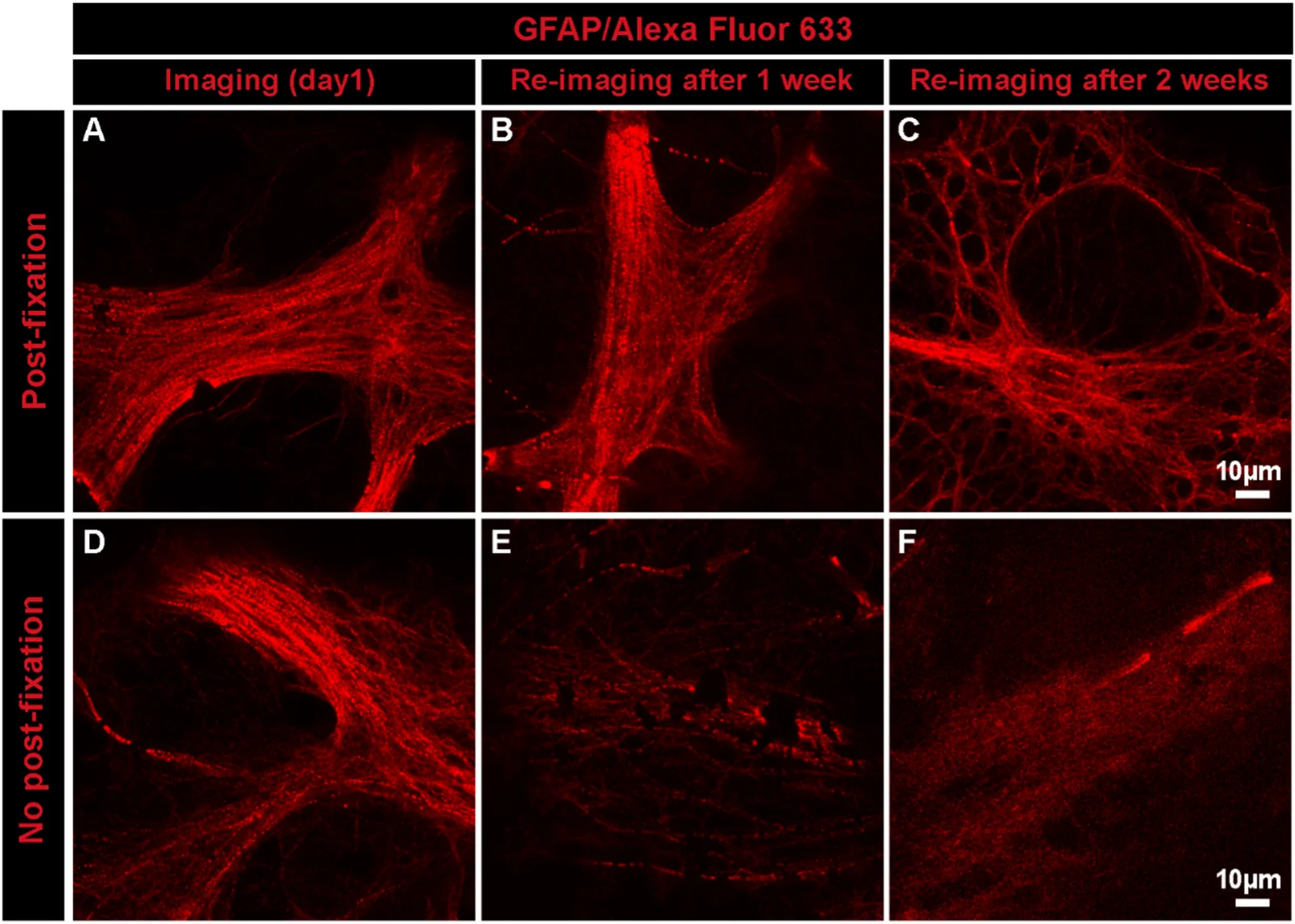
Overviews astrocytes labeled after expansion with GFAP nanobodies conjugated with Alexa Fluor 633. (**A**) Post-fixed hydrogel imaged one day after post-expansion labeling, showing distinct GFAP filamentous network, (**B**) the same post-fixed hydrogel after one week of storage at 4 °C, exhibiting typical GFAP filaments, and (**C**) the same post-fixed hydrogel re-imaged after two weeks of storage at 4 °C, demonstrating the intact GFAP filamentous network and preserved fluorescence intensity. (**D**) Non-post-fixed hydrogel imaged one day after post-expansion labeling, displaying normal GFAP filaments, (**E**) the same non-post-fixed hydrogel re-imaged after one week of storage at 4 °C, depicting a marked diminution in fluorescence intensity, and (**F**) the same non-post-fixed hydrogel re-imaged after two weeks of storage at 4 °C, revealing considerable loss of signal and structural integrity. *N* = 75 cells imaged from five independent post-fixed and non-post-fixed hydrogels

To further substantiate our findings across various cell lines, we conducted immunostainings of expanded neuron-glia primary co-cultures, relying on glial fibrillary acidic protein (GFAP), an intermediate filament protein that is often used as a marker for astrocytes. We employed direct post-labeling with GFAP nanobodies, either conjugated with Atto 542, or with Alexa Fluor 633. Upon imaging the gels, we observed specific GFAP expression in both post-fixed and non-post-fixed gels, one day after expansion. GFAP expression depicted, as expected, the filamentous intermediate filament cytoskeleton in expanded astrocytes, indicating high-quality post-expansion staining (Fig. 9A, D, and 10A, D). After one week, the post-fixed expanded astrocytes demonstrated notable GFAP signals, while non-post-fixed gels exhibited only faint signals (Figs. 9B and E and 10B and E).

Upon re-imaging the same gels after two weeks, we observed persistent and obvious GFAP signals in the post-fixed gels, accentuating the efficiency of the post-fixation process in maintaining the cytoskeletal structural integrity and signal stability (Figs. 9C and 10C). Conversely, we perceived poor GFAP signals in non-post-fixed gels, accompanied by evident degeneration of cytoskeletal architecture in the expanded astrocytes (Figs. 9F and 10F).

Collectively, post-expansion labeling of the X10 expanded gels attained high-resolution visualization of alpha tubulin and GFAP molecular structures in HEK293 and astrocyte cells, respectively, while significantly reducing issues related to label loss. Furthermore, post-expansion staining followed by post-fixation of the hydrogels using a PFA and GA mixture enhanced the stability of staining and antibody retention, enabling long-term preservation over years in HEK293. Moreover, post-fixation procedures of the hydrogels promoted the stability of staining and antibody retention over weeks in astrocytes, akin to the findings from HEK293 cells.

To further examine the universality of the post-fixation approach across different types of expansion gels, we post-fixed U-ExM gels, yielding an expansion factor of 4-5x. We immunostained expanded neuron-glia primary co-cultures, for several markers: the mitochondrial marker TOM20, the Golgi apparatus marker GM130, and the synaptic proteins synaptotagmin 1 and PSD95.

**Fig. 11:**
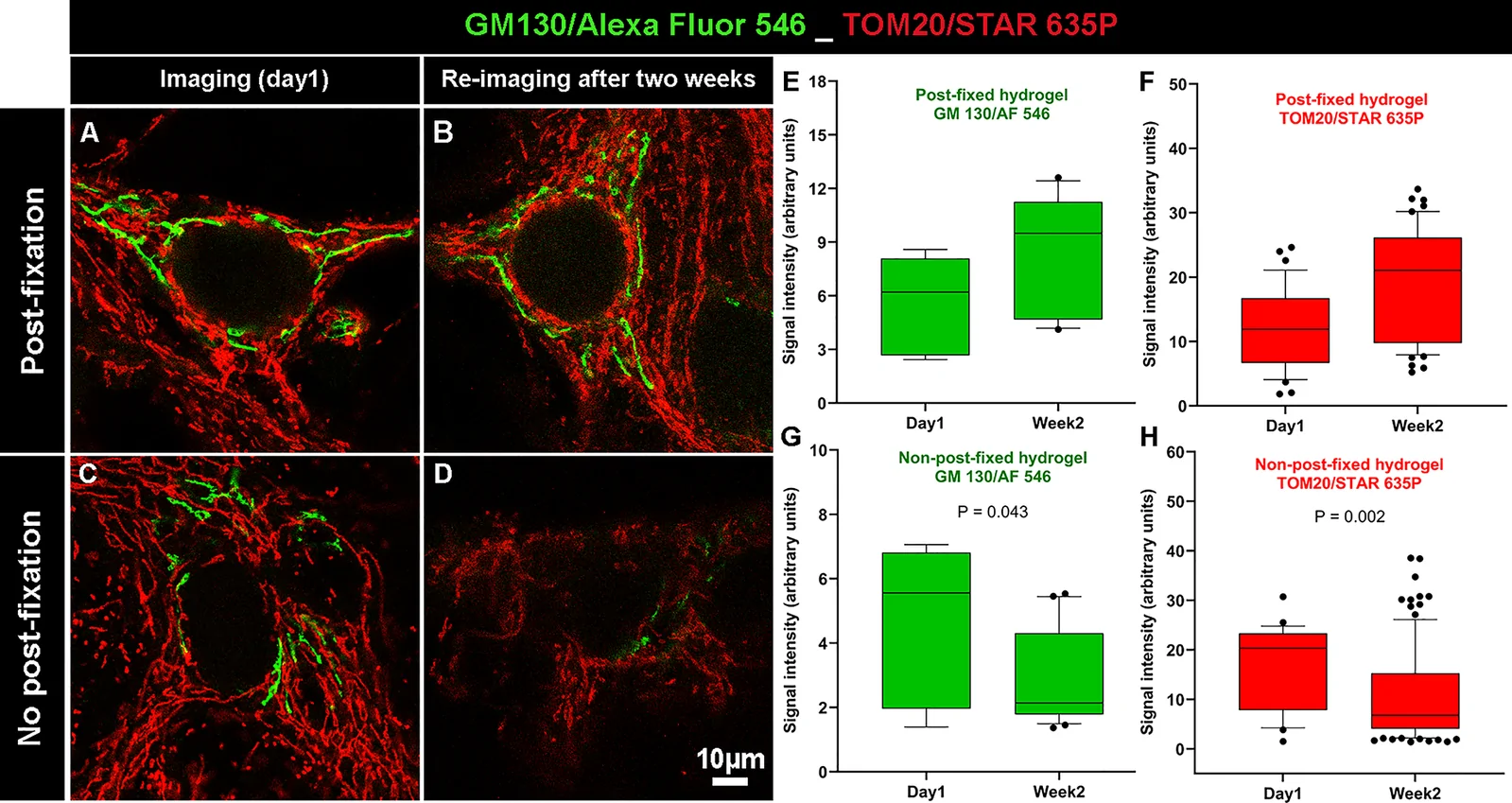
Overview of expanded neurons, following indirect post-labeling for TOM20 and GM130 in U-ExM gels. (**A**) Post-fixed U-ExM hydrogel imaged one day after post-expansion labeling, showing specific mitochondrial (TOM20) and Golgi (GM130) localization, and (**B**) the same post-fixed hydrogel after two weeks of storage at 4 °C, demonstrating preserved mitochondrial and Golgi localization and fluorescence intensity. (**C**) Non-post-fixed U-ExM hydrogel imaged one day after post-expansion labeling, displaying specific mitochondrial and Golgi localization, and (**D**) the same non-post-fixed hydrogel re-imaged after two weeks of storage at 4 °C, revealing marked signal attenuation. (**E**-**H**) Quantitative analysis revealed reduced fluorescence intensity in the non-post-fixed hydrogel. TOM20: *N* = 28 cell areas for day 1, non-post-fixed; 108 for week 2, non-post-fixed; 36 for day 1, post-fixed; 52 for week 2, post-fixed. GM130: *N* = 7 cell areas for day 1, non-post-fixed; 27 for week 2, non-post-fixed; 9 for day 1, post-fixed; 13 for week 2, post-fixed. The Mann-Whitney ranksum test was employed to test signal reductions in week 2, with P values indicated in the graphs

We first used double indirect post-labeling for TOM20 and GM130, relying on secondary antibodies conjugated to STAR 635P and Alexa Fluor 546, respectively. Upon imaging the gels, one day after expansion, specific mitochondrial and Golgi localizations were detected in both post-fixed and non-post-fixed gels (Fig. 11A and C). After two weeks, confocal imaging shown in Fig. 11B and D and quantitative analysis presented in Fig. 11E-H revealed signal attenuation in the non-post-fixed gels, but not in the post-fixed gels.

To further validate the feasibility of the post-fixation method across different proteins, we performed immunostaining on expanded neurons using double direct post-expansion labeling with nanobodies against synaptotagmin 1, conjugated to STAR 63P, and against PSD95, conjugated to Cy3.

**Fig. 12:**
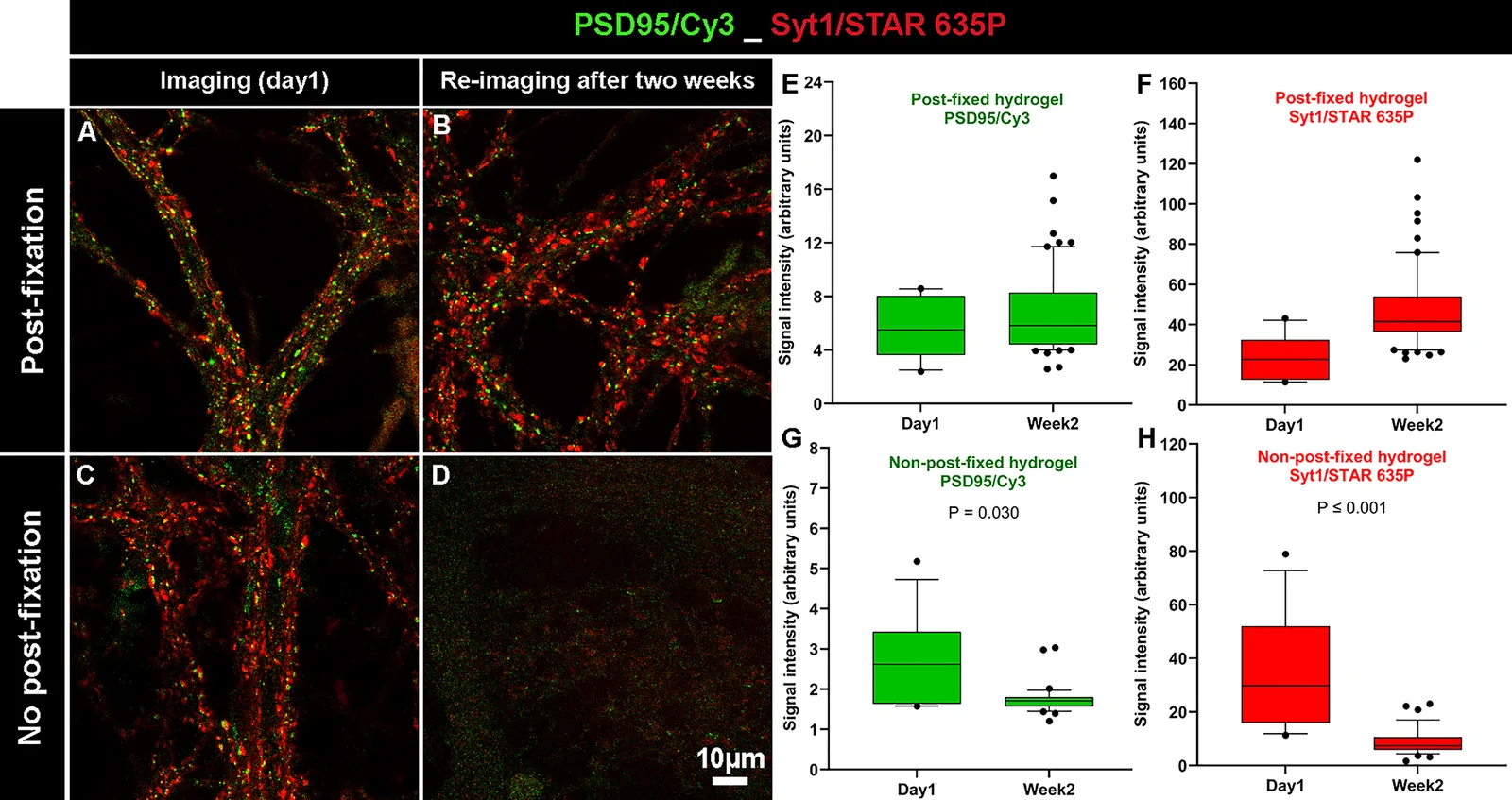
Overview of expanded neurons, following direct post-labeling with nanobodies for synaptotagmin 1 (Syt1) and PSD95 in U-ExM gels. (**A**) Post-fixed hydrogel imaged one day after post-expansion labeling, showing specific presynaptic Syt1 and postsynaptic PSD95 localization, and (**B**) the same post-fixed hydrogel after two weeks of storage at 4 °C, demonstrating retained distinct dual synaptic signals and preserved fluorescence intensity. (**C**) Non-post-fixed hydrogel imaged one day after post-expansion labeling, displaying specific presynaptic and postsynaptic localization, (**D**) the same non-post-fixed hydrogel re-imaged after two weeks of storage at 4 °C, revealing faint synaptic signals and marked signal attenuation. (**E**-**H**) Quantitative analysis confirmed reduced fluorescence intensity in non-post-fixed gels compared with post-fixed gels, underscoring the efficacy of the post-fixation approach in preserving structural and signal integrity. PSD95: *N* = 12 cell areas for day 1, non-post-fixed; 39 for week 2, non-post-fixed; 10 for day 1, post-fixed; 60 for week 2, post-fixed. Syt1: *N* = 12 cell areas for day 1, non-post-fixed; 39 for week 2, non-post-fixed; 10 for day 1, post-fixed; 60 for week 2, post-fixed. The Mann-Whitney ranksum test was employed to test signal reductions in week 2, with P values indicated in the graphs

As shown in Fig. 12A and C, confocal images of the gel, one day post-expansion revealed specific synaptic expression patterns of the presynaptic synaptotagmin 1 and postsynaptic PSD95, in both post-fixed and non-post-fixed gels. Images of the gels after two weeks showed that the post-fixed expanded neurons retained distinct synaptic signals for both markers, underscoring the capacity of the post-fixation approach to sustain structural and signal integrity (Fig. 12B). In contrast, the non-post-fixed gels displayed only faint signals, indicating instability in the conventional post-labeling protocol (Fig. 12D). Furthermore, quantitative analysis corroborated the signal reduction observed in the non-post-fixed gels (Fig. 12E-H). Overall, the integration of post-expansion labeling and post-fixation in DMAA-based and U-ExM hydrogels enhanced staining stability and antibody or nanobody retention over time.

## Discussion

It is recognized that the nature of the employed hydrogels is a key aspect for the mechanical stabilization of expansion hydrogels. Among the various types of superabsorbent gels, acrylamide/bis-acrylamide-crosslinked hydrogels and DMAA-based hydrogels are widely used in expansion protocols. Of particular interest, DMAA-based hydrogels possess a unique property arising from the dual function of DMAA as a backbone and self-crosslinker of the gel, developing strong integrity gels with high resilience against mechanical distortions [9, 10, 13]. Additionally, DMAA hydrogels emerged to exhibit remarkable mechanical characteristics in swollen state compared to acrylamide/bis-acrylamide-crosslinked hydrogels [13]. Despite the robustness of the DMAA hydrogels stemming from its intrinsic structure, it cannot prevent the label loss issue that frequently occurs during the expansion workflow when the labeling of the cells is applied before gelation [5, 15].

To reduce fluorescent label loss, we completely shifted the staining process after expansion of the hydrogels, achieving a 10x expansion factor. Therefore, we established a novel X10 post-expansion approach combined with a post-fixation step to ensure antibody retention and long-term fluorophore stability. This approach could only be achieved by slicing expanded gels into small pieces. Notably, the incised parts of the respective hydrogels retain their inherent mechanical attributes, thereby sustaining the morphological integrity and structure of the cells and enabling efficient post-labeling steps without any detectable distortions in the gels. Furthermore, this enhances the accessibility of antibodies to the epitopes of expanded cells in all directions, thereby amplifying signal intensities.

Additionally, prior studies employed post-expansion labeling of the specimens as a reasonable solution to preserve the epitopes and minimize the fluorescence loss through the homogenization, polymerization and expansion phases [6, 19]. However, our findings demonstrated that, while post-expansion labeling enables high-quality visualization of protein patterns, it is constrained by a limited short-term window for imaging. Therefore, delayed imaging beyond this temporal window could result in a rapid signals decrease, thereby losing structural information. To address this obstacle, we applied a post-fixation strategy using a mixture of PFA and GA to enhance the sample stability and antibody retention, which was effective for extended sample storage and imaging times.

We employed a combination of PFA and GA as a post-fixative agent, since the individual application of PFA often results in incomplete fixation, due to its slow penetration into samples [22, 23]. In addition, although previous reports applied a PFA/GA mixture only onto cells, aside of hydrogels, they demonstrated that this mixture enhanced fixation by facilitating faster penetration and reducing molecular mobility through improved protein crosslinking [22, 23]. We selected the GA concentration of 1%, as we found this concentration to be sufficient for fluorescence experiments, for which even 0.2% GA is entirely appropriate [21], although higher concentrations are typically used in electron microscopy [24]. To enhance structural preservation with immunolabeling compatibility in this study, we leveraged the synergistic properties of 4% PFA and 1% GA to promote fixation quality, while maintaining epitope accessibility. This post-fixation treatment may develop crosslinking bonds between the polymerized hydrogels, or proteins within the hydrogels, and the antibodies, resulting in antibody retention and stain stability. At the same time, the fixatives may alter the gel matrix directly. However, the swelling profiles of the hydrogels, as an indicator of changes in the physicochemical properties of the gel matrix, showed no significant differences in swelling behavior or expansion factor for the DMAA-based gels, implying no alteration of the gel matrix.

Remarkably, our findings demonstrated that applying the post-fixation strategy across different gel types, specifically DMAA-based and acrylamide/bis-acrylamide-based hydrogels, led to consistent signal retention enhancement. Accordingly, both hydrogel types retained fluorescent signals from fluorophore-conjugated antibodies after PFA/GA post-fixation. These observations substantiate the universality of the post-fixation protocol in enhancing signal stability. In addition to post-fixation, antioxidants (e.g., ascorbic acid and/or Trolox) could be added to storage buffers, to mitigate damage to both antibodies and fluorophores.

This innovative approach could significantly contribute to preserving the samples for long-term storage, while maintaining the structural integrity of the examined proteins, thereby advancing the potential implementation for extended analysis in pathological and medical studies. For the specific characterization of various cells lines, it is noteworthy that the stability of hydrogel samples is crucial for obtaining global overviews of expanded cells and organelles, which requires scanning many Z-stacks to obtain complete information about their architecture. This technique of imaging is also highly relevant in studies focusing on cellular differentiation and in identifying various kinds of cellular subtypes, based on their molecular markers and morphology, such as different interneurons subtypes. However, it is advisable to test the stability extent of the desired fluorophore, using either direct or indirect post-labeling, before engaging into extensive studies. Moreover, further material science investigations of the hydrogel could be conducted in future investigations, to elucidate the mechanisms used by post-fixation to preserve antibody signals.

Overall, the integration of post-fixation and post-labeling approaches reinforces the labeling and imaging efficiency of the hydrogels, over time. This enhances the visualization of cells using a simple fluorescence microscope using objective lenses with low and high magnification power, in addition to super-resolution microscopes, resulting in years of stabilized hydrogels.

## Data Availability

All the data are provided in the manuscript and further inquiries are available from the corresponding authors upon reasonable request.
